# Transforming perioperative care in Brazil: challenges and opportunities for improving outcomes

**DOI:** 10.1016/j.bjane.2025.844596

**Published:** 2025-02-05

**Authors:** Luciana C. Stefani, Liana M.T.A. Azi, Andre P. Schmidt

**Affiliations:** a Hospital de Clínicas de Porto Alegre (HCPA), Serviço de Anestesia e Medicina Perioperatória, Porto Alegre, RS, Brazil; b Universidade Federal do Rio Grande do Sul (UFRGS), Faculdade de Medicina, Departamento de Cirurgia, Porto Alegre, RS, Brazil; c Universidade Federal do Rio Grande do Sul (UFRGS), Programa de Pós-Graduação em Medicina: Ciências Médicas, Porto Alegre, RS, Brazil; d Universidade Federal da Bahia (UFBA), Faculdade de Medicina, Departamento de Anestesiologia e Cirurgia, Salvador, BA, Brazil; e Hospital Universitário Professor Edgard Santos (HUPES), Salvador, BA, Brazil; f Universidade Federal do Rio Grande do Sul (UFRGS), Porto Alegre, RS, Brazil; g Santa Casa de Porto Alegre, Serviço de Anestesia, Porto Alegre, RS, Brazil; h Hospital Nossa Senhora da Conceição, Serviço de Anestesia, Porto Alegre, RS, Brazil; i Universidade Federal do Rio Grande do Sul (UFRGS), Programa de Pós-Graduação em Ciências Pneumológicas e Programa de Pós-Graduação em Ciências Cirúrgicas, Porto Alegre, RS, Brazil; j Faculdade de Medicina da Universidade de São Paulo (FMUSP), Programa de Pós-Graduação em Anestesiologia, Ciências Cirúrgicas e Medicina Perioperatória, São Paulo, SP, Brazil

Enhancing perioperative care quality is vital for strengthening overall health systems. The topics featured in this edition of the BJAN were carefully selected for their relevance to recent advancements in perioperative care delivery. Despite the numerous opportunities to apply these innovations, significant gaps remain. This editorial highlight the major challenges in improving perioperative care and advocates for transforming perioperative medicine into a public health priority in Brazil.

The first challenge is to recognize perioperative care as a public health issue. Surgical interventions address approximately one-third of the global disease burden, and with population growth and aging demographics, expanding access to surgical services is becoming increasingly urgent.^1^ A recent study in the UK underscored this need, revealing that 60% of individuals undergo surgery at some point in their lives.^2^ Nevertheless, surgeries are not harmless. Even a single complication after surgery can have a profound impact on long-term survival.^3^ Postoperative complications experienced within 30 days following surgery cause a 1-year mortality rate to increase by two-fold.^4^ Therefore, any increase in surgical capacity must be accompanied by substantial investment that prioritizes care quality and minimizes adverse postoperative outcomes.^5^

While intraoperative safety has improved significantly, progress in mitigating the postoperative burden has been slower. Postoperative mortality is currently the third leading cause of death worldwide, and postoperative morbidity remains a significant global health challenge.^6^ Patients who develop complications often experience reduced functional independence and diminished productivity, which hinder economic growth.^1^ This underscores the importance of addressing surgical outcomes not only for individual patient well-being but also for broader societal and economic considerations. To maximize the potential of perioperative medicine, we must consider the patient's entire trajectory, focusing on value-based care and population health.^7^ As the world's fifth most populous country and the only country with over 100 million inhabitants with universal health access, Brazil faces profound inequalities between its fragmented public and private health sectors.^8^ Access problems for tertiary care result in advanced surgical diseases in most public hospitals, affecting patient risks and increasing the number of urgent procedures. Geographic workforce distribution disparities and uneven regional investments negatively impact primary care assistance and timely and safe access to surgery.^9^ This global and national perspective highlights the need for collaborative efforts and knowledge sharing across healthcare systems to identify how major health determinants, including social economic factors and structural factors impact perioperative outcomes and best practices, and to explore innovative solutions to address these problems.

The second challenge is to ask the right questions about perioperative care. We must move beyond echoing evidence-based medicine from other regions and populations without generating local research-based evidence. We should look up to countries that are setting perioperative research priorities, aiming to define where efforts should start and concentrate.^10^, ^11^, ^12^, ^13^ National research initiatives are now beginning to address the gap in perioperative data. In this issue of BJAN, a study on the effect of the pandemic on general surgical mortality highlights the role of disrupted perioperative processes in outcomes. The substantial number of additional deaths among non-COVID-19 patients suggests that the pandemic significantly disrupted surgical services in a major Brazilian university hospital, demonstrating the vulnerability of fragile surgical systems to external stressors and the urgent need for measures to increase performance and resilience. A recent study by Morais et al.^14^ showed a significant reduction in intraoperative cardiac arrest and associated 30-day mortality over the last 18 years in Botucatu, SP. Furthermore, a national risk model was recently validated in ten major hospitals to identify high-risk surgical patients with a high probability of postoperative death,^15^ grounding a high-risk pathway, and helping bedside decisions regarding the level of perioperative care.^16^ In addition, to obtain robust Latin American data, many Brazilian hospitals recently joined the Latin American Surgical Outcome Study (LASOS) initiative,^17^ which aimed at generating data to identify the surgical profile, outcomes, and key areas for future research on this topic.^5^ These studies and many others demonstrate that we can obtain large, robust data in Brazil; nevertheless, the process by which we achieve this needs to be optimized.

This is our third challenge, building more granular, nationally reported, accessible, and connected data. Numerous obstacles have hindered the widespread use of data to improve assistance in Brazil. Each hospital may adopt its own information system, whether in the public or private sector, which complicates the efforts to unify reliable information. The Brazilian National Data System (DataSUS) is constantly being improved; however, as the current hospitals’ informational systems, it is not designed to provide granular information about individual surgical patients. Consequently, there is a disconnect between what is captured by these management systems and what needs to be measured to inform care providers and stakeholders about the efficiency of perioperative assistance.

The fourth and most difficult challenge is to use reliable information to improve the quality of perioperative care. While access to and preparedness of surgery are centralized problems to be addressed in Brazil, taking care of patients once they are in hospitals can be a straightforward strategy to improve outcomes. Studies analyzing the failure-to-rescue concept have shown that postoperative deterioration is almost always gradual, with nursing staff documenting the need for care escalation.^18^^,^^19^ Consequently, failure-to-rescue (failure in the scaling of care for patients with complications) reflects hospital performance, and progressive deterioration is an opportunity to identify, intervene, and potentially preserve patients' lives.

However, given the shortage of ICU beds for the growing population of elderly and comorbid surgical patients, it is crucial to develop, adapt, and test alternatives to enhance vigilance and improve care pathways. Potential solutions may include adopting high-acuity postoperative units,^20^ developing initiatives to enhance care for high-risk patients in surgical wards,^16^^,^^21^^,^^22^ or establishing perioperative pathways for critical procedures, such as surgical laparotomies.^23^ The common sense is that the approaches to implementing improvements differ, and the obstacles are substantial.^24^ There will be no single or universal solution to address the inherent complexities of individual hospital organizations. Implementation science can help bridge the gap between what research shows as effective (evidence) and what is practiced in real-world settings, ensuring that solutions are adopted for each complex health environment.

The final challenge lies in promoting a cultural shift in perioperative care. Our focus cannot remain solely on the intraoperative period, as our specialty encompasses much more. We must strive for optimal perioperative care to positively impact our global health system while maintaining consistent standards, regardless of patients' socioeconomic status or geographic location.

In conclusion, addressing the challenges in perioperative care requires a comprehensive and transformative approach. It is imperative to advocate for a cultural shift that recognizes perioperative care as a critical public health priority, particularly in contexts marked by assistance inequities and the pressing need for rational resource allocation. Prioritizing robust data collection within a framework that integrates research into patient care can drive meaningful, system-wide improvements. By adopting these strategies, we can contribute to improving healthcare quality in Brazil, leading to better outcomes and a more equitable system for everyone.

## Conflicts of interest

The authors declare no conflicts of interest.
